# Identification of Hydroxylation Enzymes and the Metabolic Analysis of Dihydromyricetin Synthesis in *Ampelopsis grossedentata*

**DOI:** 10.3390/genes13122318

**Published:** 2022-12-09

**Authors:** Shuai Zhang, Song Gao, Yu Chen, Sha Xu, Shiqin Yu, Jingwen Zhou

**Affiliations:** 1 Science Center for Future Foods, Jiangnan University, 1800 Lihu Road, Wuxi 214122, China; 2 School of Biotechnology and Key Laboratory of Industrial Biotechnology, Ministry of Education, Jiangnan University, 1800 Lihu Road, Wuxi 214122, China; 3 National Engineering Laboratory for Cereal Fermentation Technology, Jiangnan University, 1800 Lihu Road, Wuxi 214122, China; 4 The Key Laboratory of Carbohydrate Chemistry and Biotechnology, Ministry of Education, Jiangnan University, 1800 Lihu Road, Wuxi 214122, China

**Keywords:** cytochrome P450, cytochrome P450 reductase, dihydromyricetin, flavonoid 3′,5′-hydroxylase, expression analysis

## Abstract

*Ampelopsis grossedentata* leaves are highly rich in dihydromyricetin. They have been used to make tea in China for centuries. Dihydromyricetin has many potential applications in foods and medicine. This are because it has five phenolic hydroxyl groups. However, the hydroxylases involving the biosynthesis of dihydromyricetin have not been identified and characterized. In this study, a series of hydroxylases genes, including flavanone 3-hydroxylase (F3H), flavonoid 3′-hydroxylase (F3′H), flavonoid 3′,5′-hydroxylase (F3′5′H), and cytochrome P450 reductase (CPR), were identified after RNA sequencing. The full-length CDSs of *AgF3H*, *AgF3′H*, *AgF3′5′H*, and *AgCPR* genes were amplified from the cDNA library of leaves. The aforementioned enzymes were expressed and verified in *Saccharomyces cerevisiae*. Through the substrate specificity assay, the functional *Ag*F3′H, *Ag*F3′5′H, and *Ag*CPR in *A. grossedentata* were identified. The dihydromyricetin hydroxylation process in *A. grossedentata* was successfully identified. We found that substantial carbon flux occurred through the Naringenin (NAR)–Eriodictyol (ERI)–Dihydroquercetin (DHQ)–Dihydromyricetin (DHM) and NAR–Dihydrokaempferol (DHK)–DHQ–DHM pathways. This study provides some reference for the development and utilization of the germplasm resources and molecular breeding of *A. grossedentata*.

## 1. Introduction

Flavonoids are a large class of polyphenol-rich substances found in plants [1]. They exhibit excellent anti-inflammatory and antibacterial effects and have great potential for clinical use. They can be divided into flavonoids, flavonols, dihydroflavonols, flavanones, isoflavonoids, and anthocyanins based on their structure [2]. Among these, dihydroflavonols include dihydrokaempferol (DHK) [3], dihydroquercetin (DHQ) [4], and dihydromyricetin (DHM) [5]. The development of multi-omics technology [6], high-throughput screening technology [7,8] and artificial intelligence [9] in recent years has significantly promoted the development of microbial synthesis of plant natural products such as flavonoids. Lv et al. [10] analyzed the metabolic pathway of silybin and the key enzyme ascorbic acid peroxidase 1 (Apx1) based on multi-omics technology. Xing [11] and Li [12] identified and cloned the flavonoid 3′,5′-hydroxylase (F3′5′H) from *Myrica rubra* and *Jasminum sambac* based on RNA sequencing (RNA-Seq).

*A. grossedentata* is a plant historically planted in southwestern China. It has been used for medicinal and tea-making purposes for centuries [13]. The young leaves and stems of *A. grossedentata* have been found to be rich in DHM, with a DHM content of up to 35% in young leaves [14]. As a flavonoid, DHM has good anti-inflammatory [15], antibacterial [16], anti-obesity [17], anti-oxidation [18], anti-tumor [19], and hypoglycemic effects [20], and has shown great medical and commercial values. Thus, the identification of native DHM biosynthesis pathway in *A. grossedentata* is essential to the development and utilization of the germplasm resources and molecular breeding.

The flavonoid metabolic pathway and related genes of *A. grossedentata* have been studied by several researchers in recent years. Li et al. analyzed the transcriptome of *A. grossedentata* for the first time and predicted some genes related to flavonoid synthesis based on the Kyoto encyclopedia of genes and genomes (KEGG) and other databases [21]. Yu et al. [22] also predicted several candidate genes involved in the DHM biosynthesis pathway. Li and Yu performed solid preliminary studies; however, all key genes were predicted instead of verified in vitro or in vivo. The key genes related to DHM biosynthesis, F3′H and F3′5′H, are members of the cytochrome P450 enzyme family and required the participation of cytochrome P450 reductase (CPR) and NADPH [23]. The P450 and CPR genes need anchoring to the endoplasmic reticulum membrane surface to perform their functions. Hence, the aforementioned genes need to be expressed in *S. cerevisiae* for functional verification. Moreover, the *Ag*F3′H and *Ag*F3′5′H showed unprecise substrate specificity; they might catalyze the formation of naringenin (NAR), eriodictyol (ERI), DHK, and DHQ at the 5′ carbon atom [11]. The substrate specificity of enzymes can reveal the native DHM biosynthesis pathway flux in *A. grossedentata*.

In this study, different tissues of *A. grossedentata* were selected as experimental materials. The full-length CDSs of DHM biosynthesis-related key genes, especially *AgF3′5′H*, were identified and amplified by comparative transcriptome analysis. Then, the aforementioned genes were expressed to verify its function and optimal substrates in *S. cerevisiae*. Furthermore, the process of hydroxylation of NAR to DHM was analyzed. This study provides some reference for the development and utilization of the germplasm resources and molecular breeding of *A. grossedentata*.

## 2. Materials and Methods

### 2.1. Chemical Compounds, Standards and Plant Samples

Standards, including NAR, DHK, ERI, DHQ, and DHM, were purchased from Baoji Herbest Bio-Tech Co., Ltd. (Baoji, China).

### 2.2. Genes, Plasmids and Strains

The genes used in the present study are listed in Table 1. The DNA fragment ligation was performed referring to the study by Gibson et al. [24]. *Escherichia coli* (JM109) is an ideal strain for extracting high-quality plasmid DNA. It was used for plasmid cloning and propagation. Shuttle plasmid pY26 was used for plasmid amplification and gene expression. The lithium acetate transformation method [5] was used for *S. cerevisiae* transformation. The modified strain *S. cerevisiae* C800 was used for gene expression because it does not require galactose to induce GAL promoter to express genes. The strains used in the present study are listed in Table S1.

### 2.3. Plant Cultivation and RNA-Seq

*A. grossedentata* was planted in an artificial climate chamber (Temperature, 26 °C; Light: sunlight) (Science Center for Future Foods, Jiangnan University). The plant samples were collected, immediately treated with liquid nitrogen, and sent to the laboratory under dry ice conditions for RNA-Seq and data analysis. After verifying DHM content in different tissues, the leaves under stages LS1, LS5, stem, and tendril were selected for RNA-Seq analysis. The root was selected as the control to RNA-Seq analysis.

### 2.4. Genes Amplification and Analyzation

Total RNA was extracted from *A. grossedentata* using Trizol (Sangon, Shanghai, China) method and PrimeScript RT reagent kit from Takara (Biomedical Technology, Beijing, China) with gDNA eraser reverse transcription reagent for RNA reverse transcription to obtain cDNA. The key genes related to DHM biosynthesis, *Ag*F3H, *Ag*F3′H, *Ag*F3′5′H, and *Ag*CPR, were amplified and analyzed according to the RNA-Seq (GENEWIZ, Suzhou, China) database. The primers (Sangon, Shanghai, China) were designed to amplify the target genes from cDNA of *A. grossedentata* (Table S2). The amplified polymerase chain reaction fragments were cloned into pY26 vector and then sequenced. The plasmids used in this study are listed in Table S3.

The amino acid sequences of *Ag*F3H, *Ag*F3′H, and *Ag*F3′5′H were deduced using SnapGene software (version 4.3.6). The rootless phylogenetic tree of genes (Supplementary File S1) was constructed using Mega software (version 7.0) by the neighbor-joining method with 500 bootstrap replicates. The tree was marked using iTOL (https://itol.embl.de/) accessed on 13 April 2022. Alphafold2 software (https://colab.research.google.com/) was used to predict the protein tertiary structure accessed on 15 April 2022. PyMol software (version 2.5.2) was used for protein structure alignment to compare structural similarity.

### 2.5. Strains Growth Media and Culture Conditions

Yeast nitrogen base (YNB) medium without amino acids was used, supplemented with 20 g/L glucose and corresponding auxotrophic factors: 50 mg/L histidine, 50 mg/L tryptophan, 50 mg/L uracil, and 150 mg/L leucine. Yeast extract–peptone–dextrose (YPD) medium comprised 10 g/L yeast extract, 20 g/L tryptone, and 20 g/L glucose. The transformants were selected on auxotrophic agar plates. The preculture transformants were grown in the YNB medium for auxotrophic *S. cerevisiae*. After 24 h of growth, 250 μL of preculture strains were inoculated into a 250-mL shake flask with 25 mL of YPD medium. Additionally, different substrates (NAR, DHK, ERI, or DHQ) at a concentration of 100 mg/L were added after growing the transformants for 24 h in YPD when the plasmids were expressed in C800, and the total fermentation time was prolonged to 72 h at 30 °C.

### 2.6. DHM Extraction from A. grossedentata

The *A. grossedentata* leaves were collected at different times (LS1, LS2, LS3, LS4, and LS5) to examine the DHM content. The whole tissues were cut from leaves in stages 1–5, roots, stems, and tendrils of *A. grossedentata* and washed using clean water. The tissues were lyophilized for 3 days and ground into a fine powder. Then, 50 mg of the ground sample was extracted in 1 mL of methanol. The samples were ultrasonically treated at room temperature for 1 h and centrifuged at 12,000 rpm for 15 min, and the supernatant was transferred to a volumetric flask. After repeated extraction for three times, the volume was fixed to 5 mL. A 0.22-μm membrane was used to filter the extract to obtain HPLC samples. We could not purchase pentahydroxyflavanone (PHF), hence we calculated PHF titer based on material conservation.

### 2.7. Quantitative HPLC Analysis

The products were analyzed using HPLC (Shimadzu Corporation, Kyoto, Japan). After fermentation in shake flasks, 500 μL of the fermentation broth was mixed with 500 μL of methanol and the mixture was centrifuged at 13,000 rpm for 3 min. The supernatant was filtered using a 0.22-μm filter membrane. The HPLC (Shimadzu Corporation) was equipped with a reverse-phase C18 column (4.6 × 150 mm, Thermo Fisher Scientific, Inc., Waltham, MA, USA) and maintained at 40 °C. The detection wavelength was set at 290 nm. A flow rate of 1.0 mL/min was used with a gradient elution method: (1) 0.1% (*v*/*v*) trifluoroacetic acid in water and (2) 0.1% (*v*/*v*) trifluoroacetic acid in acetonitrile, 0−10 min, 10−40% B; 10−20 min, 40−60% B; 20−22 min, 60%−10% B; and 22−25 min, 10% B.

### 2.8. Data Processing and Statistical Analysis

All data are represented by the mean of three biologically independent samples. The data were calculated using Excel (version 2019). The one-tailed Student *t* test and Tukey’s test were calculated by SPSS (version 22) (^ns^ *p* > 0.05, * *p* < 0.05, ** *p* < 0.01, *** *p* < 0.001). Error bars represent the mean ± standard deviation.

## 3. Results

### 3.1. Spatial DHM Content in A. grossedentata

**Figure 1:**
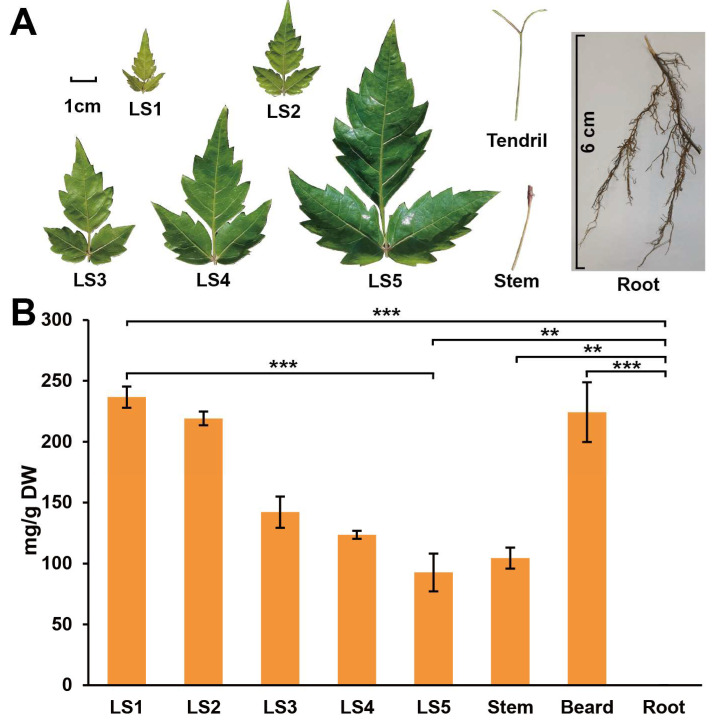
Spatial DHM content in *A. grossedentata*. (**A**) Five stages of leaves, stems, tendrils and roots. The 6-cm scale bar was used for roots and the 1-cm scale bar was used for LS1–LS5, stems, and tendrils. (**B**) Dry DHM weight contents in different tissues of *A. grossedentata* (** *p* < 0.01, *** *p* < 0.001). Abbreviations: DHM, dihydromyricetin; LS, leaf stage.

Secondary metabolites may be distributed differently in different plant tissues [14], so identifying the distribution is critical for further pathway analysis. The samples were taken from various tissues in order to further investigate the DHM distribution in *A. grossedentata* leaves. The dry DHM weight content in *A. grossedentata* leaves LS1, LS2, LS3, LS4, and LS5 was 236.64, 219.13, 142.23, 123.59, and 92.67 mg/g DW, respectively. The content in the roots, stems, and tendrils was 0, 104.41, and 224.25 mg/g DW, respectively (Figure 1). The DHM content decreased gradually with the increase in the age of leaves. The DHM content in *A. grossedentata* had obvious temporal and spatial differences (Figure 1B).

### 3.2. Analysis of Dihydroflavonol Metabolic Pathway and Genes Expression in A. grossedentata

**Figure 2:**
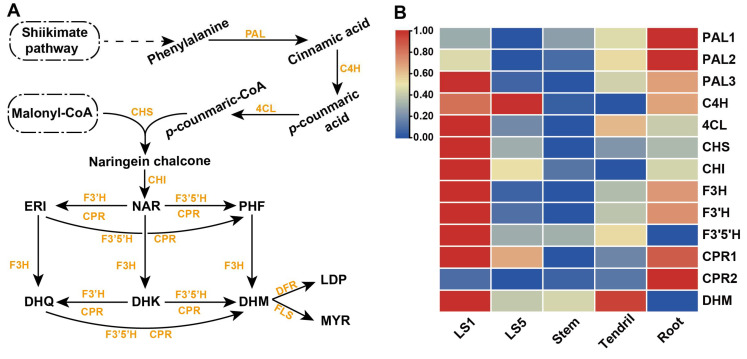
DHM synthesis pathway and analysis of gene expression of each sample. (**A**) Orange and black letters indicate enzymes and compounds, respectively. Abbreviations: DHK, Dihydrokaempferol; DHM, Dihydromyricetin; DHQ, Dihydroquercetin; ERI, Eriodictyol; NAR, Naringenin; MYR, Myricetin; LDP, Leucodelphinidin. (**B**) The gene expression profile in different tissues was shown in heat map, and each gene in different tissues was shown in a row and was normalized to the highest expression level in this row. The row represents each sample of *A. grossedentata*, and the list shows the genes related to flavonoid synthesis in *A. grossedentata.* Red, yellow, and blue rectangles represent the change in the relative expression of each column from high to low.

DHM synthesis was mainly divided into two modules (Figure 2A). Phenylalanine generated via the shikimic acid pathway was catalyzed by phenylalanine deaminase (PAL), cinnamate 4-hydroxylase (C4L), 4-cinnamate coenzyme A ligase (4CL), chalcone synthase (CHS) and chalcone isomerase (CHI) to produce NAR. Then NAR thrice underwent hydroxylation of flavanone 3-hydroxylase (F3H), flavanone 3′-hydroxylase (F3′H), and flavanone 3′,5′-hydroxylase (F3′5′H) to generate DHM.

The RNA-Seq analysis revealed many differentially expressed genes in the roots and leaves (LS1 and LS5), as well as the stems and tendrils (DEGs) (Figure S1). KEGG enrichment analysis results showed that DEGs were mainly enriched in metabolism-related metabolic pathways and secondary metabolism (Figure S2). This study focused on DHM biosynthesis; therefore, we were interested in the flavanone metabolic pathway enrichment genes. The number of flavonoid biosynthesis enzymes obtained via KEGG enrichment analysis (Figure S2) among groups was 53, 50, 32, and 46. After sorting out the length of the aforementioned sequences, 12 flavone metabolic pathway genes were obtained (Table 2). One gene each of *Ag*F3H, *Ag*F3′H and *Ag*F3′5′H and two *Ag*CPR genes were obtained.

The analysis of gene expression level (Figure 2B) showed that all genes (except *PAL1*, *PAL2*, and *CPR2*) were highly expressed in LS1. The hydroxylation genes had different expression levels in different tissues. The expression levels of *AgF3H* and *AgF3′H* decreased from LS1, to tendrils, to LS5, and to stems. *AgF3′5′H* had the highest expression in both LS1 and tendrils. The expression intensity of *AgF3′5′H* in each sample decreased from LS1, to tendrils, to stems, and to LS5, which followed the same trend as the DHM content. Although the roots did not contain DHM, the expression of *AgF3H*, *AgF3′H*, and *AgCPR1* was relatively high.

### 3.3. Amplification and Analysis of Key Genes Related to DHM Biosynthesis

**Figure 3:**
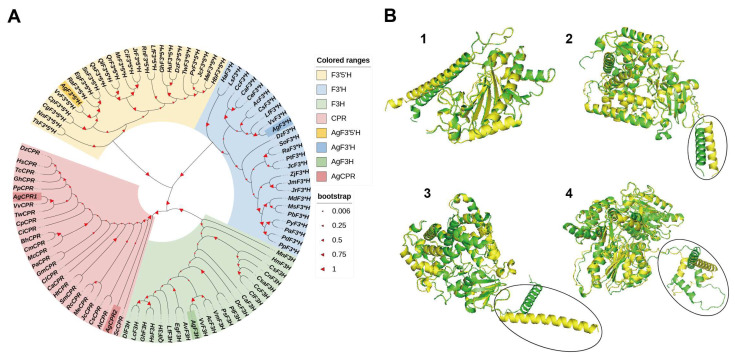
Phylogenetic analysis of NAR hydroxylase and tertiary structure prediction. (**A**) Red, green, yellow, and blue represent F3H, F3′H, F3′5′H and CPR from different sources, respectively, and corresponding dark colors represent *Ag*F3H, *Ag*F3′H, *Ag*F3′5′H and *Ag*CPR. (**B1**) Yellow one was *Ag*F3H, and the green represents *Cs*F3H. (**B2**) Yellow one represents *Ag*F3′H, and the green represents *Sm*F3′H. (**B3**) Yellow one represents *Ag*F3′5′H, and the green represents *Sl*F3′5′H. (**B4**) Yellow one represents *Ag*CPR1, and the green represents *Sm*CPR. Transmembrane domains are shown within the circle. Alphafold2 was used to predict the protein tertiary structure and then PyMol software (New York, NY, USA) was used for protein tertiary structure alignment.

The full-length CDS of AgF3H was 1122 bp and encoded 373 amino acids. *AgF3′H* had a total length of 1530 bp and encoded 509 amino acids. *AgF3′5′H* had a total length of 1527 bp and encoded 508 amino acids. *AgCPR1* and *AgCPR2* had a total length of 1530 bp and encoded 509 amino acids. The phylogenetic evolution analysis results are shown in Figure 3A. *AgF3H*, *AgF3′H*, *AgF3′5′H*, and *AgCPRs* were clustered in four different branches. *A. grossedentata* and Vitis vinifera belonged to the family Vitaceae, and their homology was the most similar. However, the genetic relationship between *Ag*CPR2 and *Ag*CPR1 was not the closest. The expression of *Ag*CPR2 showed no difference at the transcriptome level of each sample, and the expression was extremely low (Table S4). The tertiary structure of enzymes (Figure 3B) identified from *A. grossedentata* were similar to that of the isozyme used in DHM synthesis [5]. These results indicated that these genes are responsible for DHM synthesis.

### 3.4. Comparison of AgCPRs and CPRs from Previous Studies

**Figure 4:**
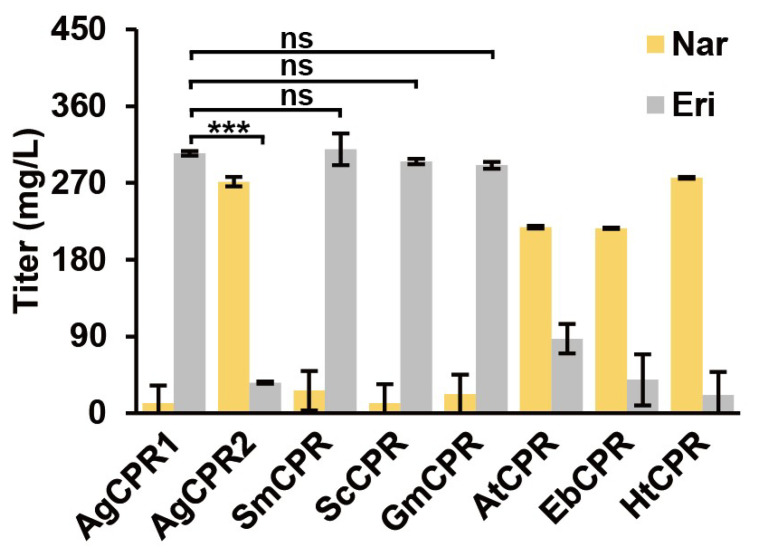
Comparison of AgCPR and CPRs from previous studies. All data are represented by the mean of three biologically independent samples (ns *p* > 0.05, *** *p* < 0.001). Abbreviations: ERI, Eriodictyol; NAR, Naringenin.

Li et al. showed different effects of the species of CPR on F3′5′H [5]. Different CPRs in combination with *Ag*F3′H were expressed in yeast to compare their function. The functional activity of CPRs, as well as their suitability with *Ag*F3′H, was evaluated via the ERI content generated by catalytic 500 mg/L NAR. The results indicated that *Ag*F3′H exhibited different activities with CPRs of different origins. *Ag*CPR1, *Ag*CPR2, *Sm*CPR, *Sc*CPR, *Gm*CPR, *At*CPR, *Eb*CPR, and *Ht*CPR assisted *Ag*F3′H in transforming NAR to obtain ERI, which were 304.59, 35.85, 309.47, 295.19, 290.56, 87.45, 39.43, and 21.56 mg/L, respectively (Figure 4). *Ag*F3′H showed strong activity in combination with cognate *Ag*CPR1, heterologous *Sm*CPR, *Sc*CPR, and *Gm*CPR. However, it showed poor activity when combined with homologous *Ag*CPR1 and heterologous *At*CPR, *Eb*CPR, and *Ht*CPR. Although both *Ag*CPR1 and *Ag*CPR2 were derived from *A. grossedentata*, *Ag*CPR2 had low suitability with *Ag*F3′H compared with *Ag*CPR1.

### 3.5. AgF3H and AgF3′H Functional and Efficiency Validation

**Figure 5:**
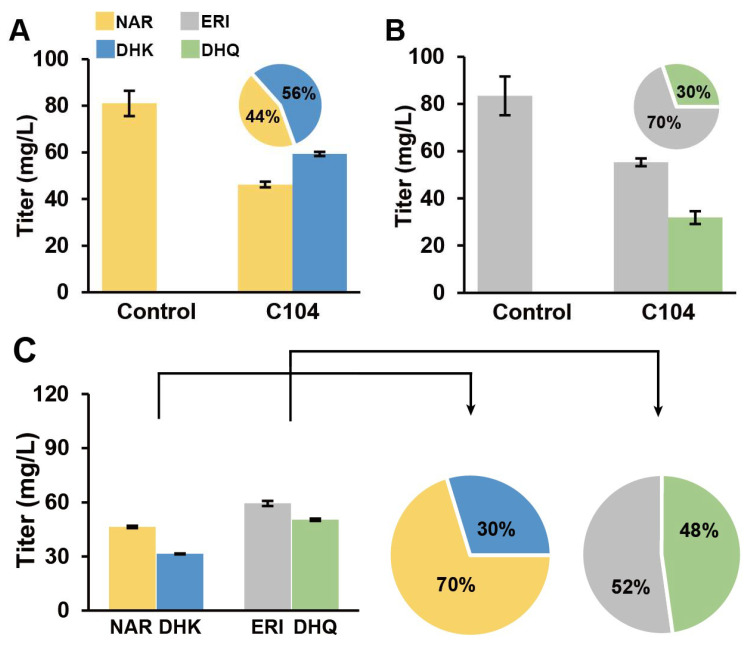
*Ag*F3H functional validation and substrates conversion efficiency analysis. (**A**) *Ag*F3H catalyzed the conversion of NAR into DHK. (**B**) *Ag*F3H catalyzed the conversion of ERI into DHQ. (**C**) *Ag*F3H simultaneously catalyzed NAR and ERI to generate DHK and DHQ. The bar graph shows the material titer in the fermentation broth. The pie chart shows the molar conversion rate of the converted product. Abbreviations: DHK, Dihydrokaempferol; DHM, Dihydromyricetin; DHQ, Dihydroquercetin; ERI, Eriodictyol; NAR, Naringenin.

When only one substrate (NAR or DHK) was added to the medium, *Ag*F3H catalyzed the formation of 59.33 mg/L DHK from 100 mg/L NAR and 31.89 mg/L DHQ at 100 mg/L ERI. The molar conversion was approximately 56% and 30% (Figure 5A,B). NAR and ERI coexist in *A. grossedentata* cells, and hence *Ag*F3H can catalyze the generation of DHK and DHQ. When the aforementioned two substrates were added at the same time to simulate the cell metabolic environment, *Ag*F3H catalyzed the formation of 29.71 mg/L DHK and 47.73 mg/L DHQ from 100 mg/L NAR and 100 mg/L ERI. The molar conversion was approximately 30% and 48% (Figure 5C).

**Figure 6:**
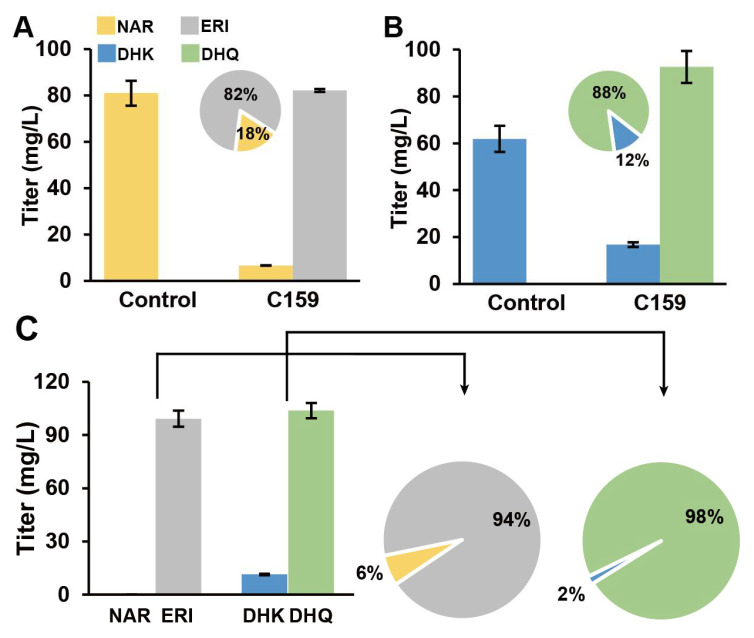
*Ag*F3′H functional validation and substrates conversion efficiency analysis. (**A**) *Ag*F3′H catalyzed the conversion of NAR into ERI. (**B**) *Ag*F3′H catalyzed the conversion of DHK into DHQ. (**C**) *Ag*F3′H simultaneously catalyzed NAR and DHK to generate ERI and DHQ, respectively. The bar graph shows the material titer in the fermentation broth. The pie chart shows the molar conversion rate of the converted product. Abbreviations: DHK, Dihydrokaempferol; DHM, Dihydromyricetin; DHQ, Dihydroquercetin; ERI, Eriodictyol; NAR, Naringenin.

*Ag*F3′H was P450 monooxygenase, which required the collaborative expression of *Ag*CPR1 to realize its function. *Ag*F3′H and *Ag*CPR1 catalyzed the formation of 82.20 mg/L ERI from 100 mg/L NAR and 92.63 mg/L DHQ from 100 mg/L DHK. The molar conversion was approximately 82% and 88% (Figure 6A,B). C159 catalyzed the generation of 99.25 mg/L DHK and 103.80 mg/L DHQ from 100 mg/L NAR and 100 mg/L ERI when NAR and DHK existed simultaneously. The molar conversion was approximately 94% and 98% (Figure 6C).

### 3.6. AgF3′5′H Functional Validation

**Figure 7:**
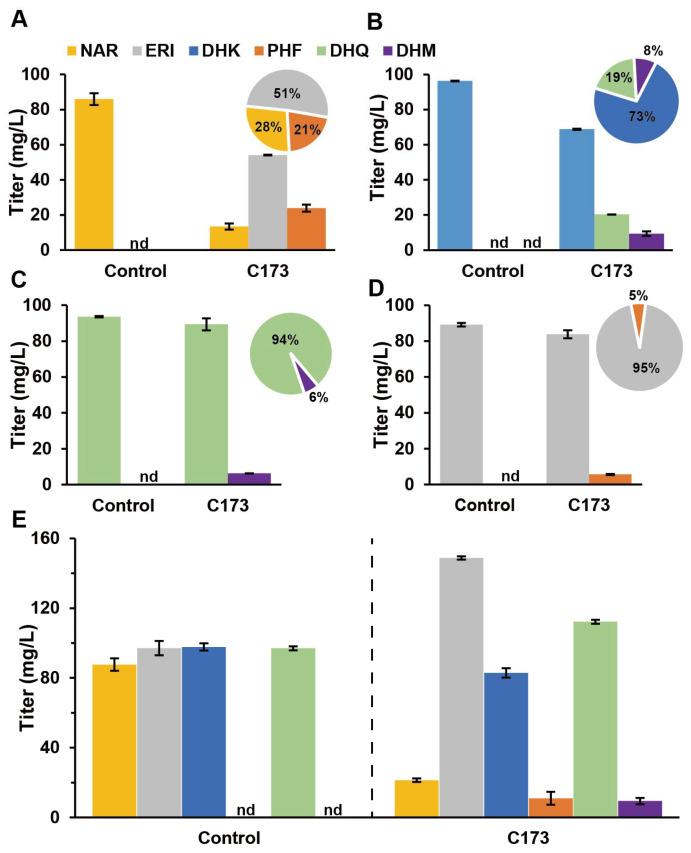
*Ag*F3′5′H functional validation and substrates conversion efficiency analysis. (**A**) *Ag*F3′5′H catalyzed the conversion of NAR into ERI and PHF. (**B**) *Ag*F3′5′H catalyzed the conversion of DHK into DHQ and DHM. (**C**) *Ag*F3′5′H catalyzed the conversion of DHQ into DHM. (**D**) *Ag*F3′5′H catalyzed the conversion of ERI into PHF. (**E**) *Ag*F3′5′H simultaneously catalyzed NAR, DHK, DHQ, and DHK to generate ERI, PHF, DHQ, and DHM, respectively. The bar graph shows the material titer in the fermentation broth. The pie chart shows the molar conversion rate of the converted product. Abbreviations: DHK, Dihydrokaempferol; DHM, Dihydromyricetin; DHQ, Dihydroquercetin; ERI, Eriodictyol; PHF, Pentahydroxyflavanone; NAR, Naringenin. nd: No corresponding compound was detected.

*Ag*F3′5′H can catalyze NAR, ERI, DHK, and DHQ to produce ERI, PFH, DHQ, and DHM, respectively (Figure 2A). Like *Ag*F3′H, we first analyzed the catalytic efficiency of a single substrate. The study results showed that C173 could catalyze the generation of 54.13 mg/L ERI and 23.92 mg/L PHF from 100 mg/L NAR (Figure 7A). Under the same conditions, 100 mg/L DHK generated 20.36 mg/L DHQ and 9.32 mg/L DHM (Figure 7B). Further, 100 mg/L DHQ was catalyzed by C173 to generate 6.24 mg/L DHM (Figure 7C). Similarly, C173 catalyzed 100 mg/L ERI to generate 5.63 mg/L PHF (Figure 7D). When 100 mg/L of each of the four substrates that *Ag*F3′5′H could act on were added to the fermentation broth, the NAR was consumed significantly, the DHK was consumed slightly, the ERI titer increased significantly to 148.76 mg/L, and the DHQ titer increased. PHF and DHM titers were detected in the culture system as 3.69 and 9.46 mg/L, respectively (Figure 7E).

## 4. Discussion

*A. grossedentata* is a unique ancient vine plant in China. Its leaves contain high levels of DHM, [14,20] and it is used for making tea [13]. Exploring its metabolic pathway and related genes for application in foods [18,26] and medicines [17,19,20,27] was of great significance. Many studies have been performed on *Ag*F3H, *Ag*F3′H, and *Ag*F3′5′H in different plants, such as *Reaumuria trigyna* [28], *Brassica rapa* [23], *Brunfelsia acuminata* [12], and *Solanum lycopersicum* [29]. However, the function of *Ag*F3′H, *Ag*F3′5′H, and *Ag*CPR in *A. grossedentata* has not been reported. In this study, a total of 12 DHM synthesis pathway genes were identified from *A. grossedentata* via RNA-Seq. *S. cerevisiae*, as a commonly used strain for the heterologous expression of CYPs [5,30,31], has many advantages over *E. coli* [32]. Therefore, *Ag*F3H, *Ag*F3′H, *Ag*F3′5′H, and *Ag*CPRs were verified in *S. cerevisiae*. Then, the native DHM biosynthesis pathway in *A. grossedentata* was confirmed based on RNA-Seq and heterologous expression in *S. cerevisiae*.

This study found that the distribution of DHM content was uneven in time and space. The DHM content decreased with the increase in the age of leaves, which was basically consistent with the findings of Li et al. [21]. However, the highest content detected in this study was only about 23% of the dry weight. This might be because DHM was different in different strains [22] and growth environments. Interestingly, the tendrils also contained a high DHM content. A higher level of *Ag*F3′5′H expression was found in tendrils, which was similar to that in young leaves. However, the expression level of other DHM biosynthesis genes in tendrils was not as high as that of LS1, like 4CL, CHI, and CHS (Figure 2B). It may not be able to synthesize enough DHM precursors by itself. Thus, we speculated that there may be some mechanism of DHM precursor transport between leaves and tendrils. However, this study was not yet able to determine a transport mechanism between tissues. The tendrils contained more DHM when they had relatively higher levels of *Ag*F3′5′H. Except *AgF3′5′H*, the expression of other genes such as *AgPALs, AgF3H,* and *AgF3′H* was relatively high in roots, but no or trace DHM was found [21] because of the low expression of *Ag*F3′5′H. High DHM content was also detected in other tissues expressing *Ag*F3′5′H. This indicated that *Ag*F3′5′H, verified in this study, was the most critical gene for DHM synthesis.

The hydroxylation of NAR was an important step in DHM biosynthesis. It was generally believed that F3′H [23] or F3H [33] was the first enzyme to guide this metabolic pathway. At the same time, F3′5′H [11,12] was the key enzyme for DHM synthesis. Li et al. [21] revealed the expression pattern and differential distribution of genes related to DHM biosynthesis through the transcriptome of *A. grossedentata*. Without considering the correctness of sequence length, they annotated 18 for *Ag*F3H, 8 for *Ag*F3′5′H, and 1 for *Ag*F3′H. Yu et al. [22] elucidated candidate genes involved in the biosynthesis and regulation of flavonoids (DHM and DHQ) through comparative transcriptome analysis. In their study, they found that the upregulation of *Ag*F3′5′H expression increased the DHQ and DHM contents. They annotated 18 for *Ag*F3H, 8 for *Ag*F3′5′H, and 1 for *Ag*F3′H. Different from their research, we set the sequences screening length of F3H, F3′H, F3′5′H, and CPR to 900, 1300, 1300, and 1800 bp. Finally, we got one for *Ag*F3H, *Ag*F3′H, and *Ag*F3′5′H, and two for *Ag*CPR, with length and protein tertiary structure similar to those of its isozymes.

**Figure 8:**
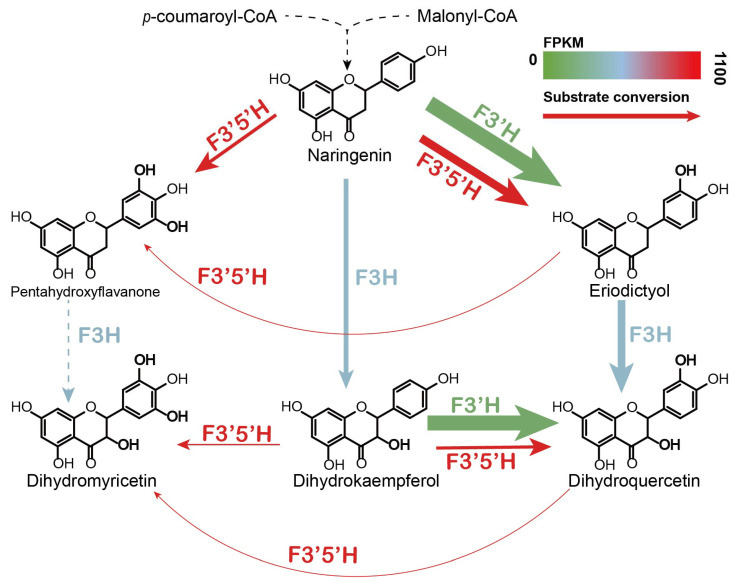
DHM metabolic pathway in LS 1. The thickness of the arrow represents the conversion rate of the corresponding substrate. The colors represent the fragment per kilo bases per million reads (FPKM) of the genes. Dashed lines represent no detection.

Although previous studies predicted the genes of *AgF3H, AgF3′H,* and *AgF3′5′H* through RNA-Seq, except for *AgCPRs* [21,22], the catalytic activity and substrate specificity of the enzymes were not confirmed. RNA-Seq analysis can quickly predict genes function, but the splicing accuracy of the next-generation sequencing fragments may not be guaranteed. Therefore, we amplified and heterogeneously expressed these key genes in *S*. *cerevisiae* and verified their substrate specificity through precursor feeding. *Ag*F3H showed higher catalytic activity for ERI in the presence of double substrates (Figure 5). *Ag*F3′H (CPR1) had higher substrate conversion efficiency and had no substrate preference (Figure 6). Relatively high ERI and DHQ titers and low PHF and DHM titers were detected in analysis of *Ag*F3′5′H (CPR1) (Figure 7). These findings indicated that the 3′ hydroxylation ability was stronger than that of the 5′, and it was not an efficient 5′ hydroxylase. This also revealed the reason for *Ag*F3′5′H expression being much higher than that of other genes. Similarly, *Ag*CHS with the highest expression level (Table S4) also had low activity [34]. The biocatalytic pathway was mapped from NAR to DHM in LS1 (Figure 8). *Ag*F3′H (CPR1) and *Ag*F3′5′H (CPR1) catalyzed the rapid transformation of NAR into ERI, and only a small amount passed through other channels. ERI was transformed into DHQ by *Ag*F3H, and a small amount of DHK was rapidly transformed into DHQ. DHQ was catalyzed to generate DHM under the catalysis of a large amount of *Ag*F3′5′H with high expression.

In this study, we detected the distribution of DHM in time and space in *A. grossedentata* and identified a series of DHM synthesis-related genes based on transcriptomics and metabonomics data. *Ag*CHS had a high expression level in NAR synthesis pathway. This indicated that *Ag*CHS was a key enzyme providing the precursor for DHM synthesis. The expression of *Ag*F3′5′H was correlated with the spatial and temporal distributions of DHM. The expression of *Ag*F3′5′H in young leaves and tendrils was 6.7 and 4.1 times higher than that in roots (Table S4). This may be the main reason why the young *A. grossedentata* leaves contain higher DHM content and the roots do not contain DHM. Both *Ag*F3H and *Ag*F3′H had bifunctional enzyme activity. The multifunctional enzyme activity of *Ag*F3′5′H was also verified, which could efficiently transform NAR into ERI. However, its weak 5′-hydroxylation activity made it difficult to catalyze the conversion of DHQ into DHM under the same expression conditions. This might be the reason why we did not obtain high titer DHM in *S. cerevisiae*. Furthermore, we also found that the catalytic activity of the enzyme was negatively correlated with its expression. From the results of *Ag*CHS and *Ag*F3′5′H activity and expression analysis, we speculated that if the transcriptional level expression of a gene in plants is much higher than that of other genes, it may not have high catalytic activity. This conclusion may have certain guiding significance for screening highly efficient enzymes from plants.

## 5. Conclusions

In this work, a comparative transcriptome analysis of *A. grossedentata* was conducted to explore the hydroxylase genes involved in dihydromyricetin biosynthesis. We constructed some *S. cerevisiae* strains to characterize the functions of the hydroxylase genes. The three functional NAR hydroxylases in *A. grossedentata* had been successfully identified and the hydroxylation metabolic pathways had been analyzed according to substrate specificity. We found that substantial carbon flux occurred through the NAR–ERI–DHQ–DHM and NAR–DHK–DHQ–DHM pathway, and less carbon flux occurred through the NAR–DHK–DHM, NAR–ERI–pentahydroxyflavanone, (PHF)–DHM, and NAR-PHF-DHM pathway. This study provides some reference for the development and utilization of the germplasm resources and molecular breeding of *A. grossedentata*. The identified enzymes also offer more selection for metabolic engineering to synthesize dihydroflavonol and anthocyanin. 

## Figures and Tables

**Table 1:** Genes used in this study.

Gene Name	Species	Source
CsF3H *	*Citrus sinensis*	MH208416
*SmF3* *′H*	*Silybum marianum*	[10]
*SlF3* *′5* ′H *	*Solanum lycopersicum*	[5]
SmCPR *	*S. marianum*	[10]
*ScCPR*	*S. cerevisiae*	CP046088.1,
GmCPR *	*Glycine max*	XM_003541568.3
AtCPR *	*Arabidopsis thaliana*	X66017.1
EbCPR *	*Erigeron breviscapus*	[25]
HtCPR *	*Helianthus tuberosus*	Z26250.1
*AgF3H*	*A. grossedentata*	This work
*AgF3* *′H*	*A. grossedentata*	This work
*AgF3* *′5* *′H*	*A. grossedentata*	This work
*AgCPR1*	*A. grossedentata*	This work
*AgCPR2*	*A. grossedentata*	This work

* represented the gene for codon optimization in *S. cerevisiae*.

**Table 2:** Genes related to DHM synthesis in *A. grossedentata*.

Gene	Unigene_ID	CDS Length (bp)
*AgPAL1*	DN94338_c1_g2_i2	2133
*AgPAL2*	DN69862_c0_g1_i1	2172
*AgPAL3*	DN94338_c1_g1_i1	2145
*AgC4H*	DN104001_c1_g1_i3	1302
*Ag4CL*	DN87908_c0_g5_i1	1497
*AgCHS*	DN99909_c4_g1_i2	1182
*AgCHI*	DN86478_c0_g1_i1	717
*AgF3H*	DN93644_c0_g1_i1	1092
*AgF3* *′H*	DN153979_c0_g1_i1	1530
*AgF3* *′5* *′H*	DN100751_c3_g1_i1	1527
*AgCPR1*	DN94781_c0_g1_i1	2118
*AgCPR2*	DN96223_c0_g1_i2	1989

## Data Availability

The data presented in this study are available on request from the corresponding author.

## Supplementary Materials

The following supporting information can be downloaded at: https://www.mdpi.com/article/10.3390/genes13122318/s1, Figure S1: The number of differentially expressed genes among control groups; Figure S2: Metabolic pathway enrichment analysis of differentially expressed genes; Table S1: *S. cerevisiae* strains used in this study; Table S2: Primers used in this study; Table S3: Plasmids used in this study; Table S4: Fragment per kilo bases per million reads (FPKM) of different genes in every sample; Supplementary File S1: Sequences used in phylogenetic tree construction and multiple alignment.

## Abbreviations

F3H, Flavanone 3-hydroxylase; F3′H, Flavonoid 3′-hydroxylase; F3′5′H, Flavonoid 3′,5′-hydroxylase; CPR, Cytochrome P450 reductase; DHK, Dihydrokaempferol; DHM, Dihydromyricetin; DHQ, Dihydroquercetin; ERI, Eriodictyol; PHF, Pentahydroxyflavanone; NAR, Naringenin; PAL, Phenylalanine deaminase; C4L, Cinnamate 4-hydroxylase; 4CL, 4-cinnamate coen-zyme A ligase; CHS, chalcone synthase; CHI, chalcone isomerase.
