# Supplementary material for: Identification of Hydroxylation Enzymes and the Metabolic Analysis of Dihydromyricetin Synthesis in Ampelopsis grossedentata
Source: Genes (Basel). 2022 Dec 9;13(12):2318. doi: 10.3390/genes13122318 (PMC9778615; doi:10.3390/genes13122318)
Supplement: Supplementary file 1 [file genes-13-02318-s001.zip › Supplementary information 1.pdf]

**Identification of hydroxylation enzymes and metabolic analysis for dihydromyricetin synthesis in Ampelopsis grossedentata**

Shuai Zhang[1], Song Gao[1,2], Yu Chen[1,2], Sha Xu[4], Shiqin Yu[1,2], Jingwen Zhou[1,2,3,4*]

[1] Science Center for Future Foods, Jiangnan University, 1800 Lihu Road, Wuxi, Jiangsu 214122, China

[2] School of Biotechnology and Key Laboratory of Industrial Biotechnology, Ministry of Education, Jiangnan University, 1800 Lihu Road, Wuxi, Jiangsu 214122, China

[3] The Key Laboratory of Carbohydrate Chemistry and Biotechnology, Ministry of Education, Jiangnan University, 1800 Lihu Road, Wuxi, Jiangsu 214122, China

[4] National Engineering Laboratory for Cereal Fermentation Technology, Jiangnan University, 1800 Lihu Road, Wuxi, Jiangsu 214122, China

[*] Corresponding author: Jingwen Zhou

Mailing address: Science Center for Future Foods, Jiangnan University, 1800 Lihu Road, Wuxi, Jiangsu 214122, China

Phone: +86-510-85914371, Fax: +86-510-85914371

E-mail: zhoujw1982@jiangnan.edu.cn.

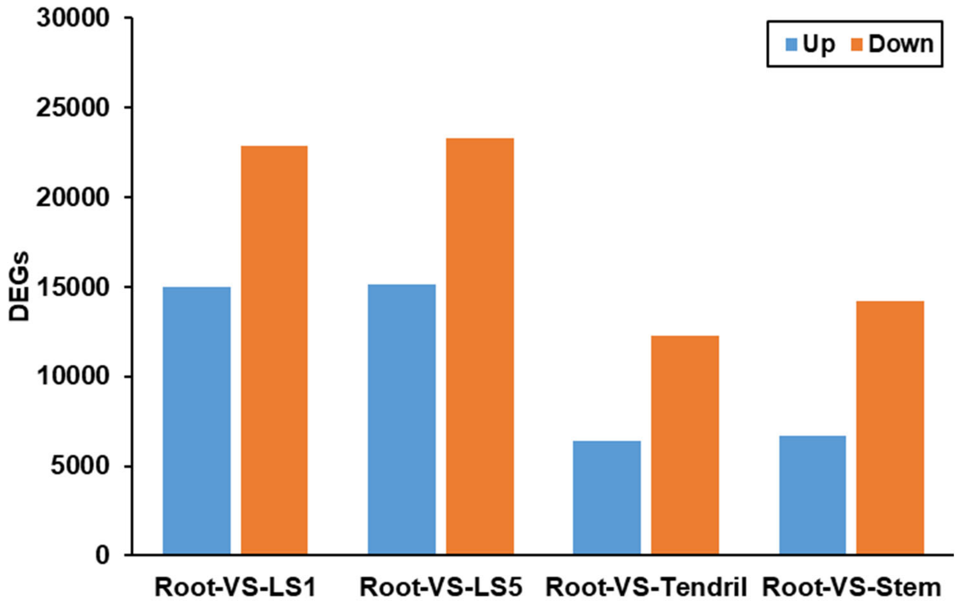


**Fig.S1. The number of differentially expressed genes among control groups.**

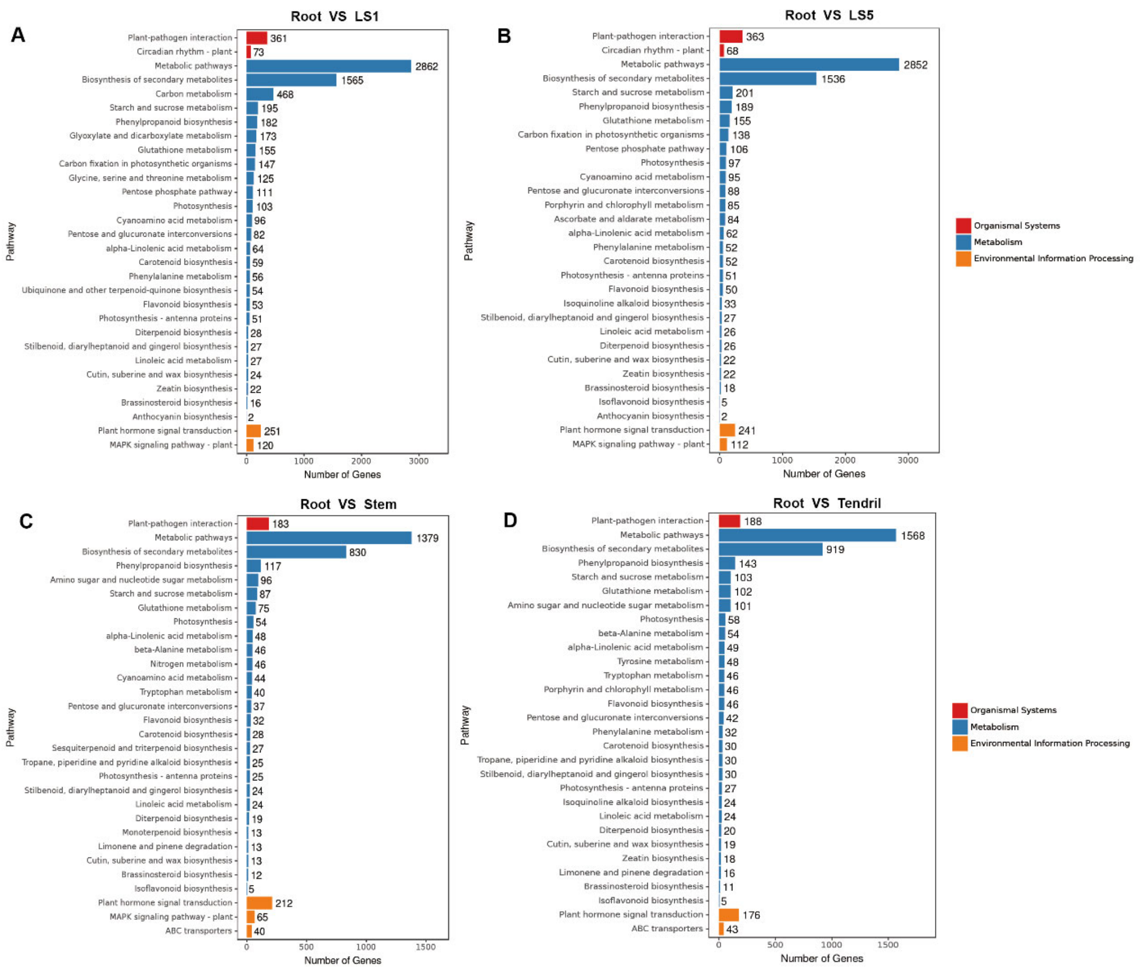


**Fig.S2. Metabolic pathway enrichment analysis of differentially expressed genes.**

The x-axis shows the total number of DEGs annotated to a given pathway. The y-axis indicates each KEGG metabolic pathway name.

**Table S1. Saccharomyces cerevisiae strains used in this study.**

| Strains | Relevant properties |
| --- | --- |
| C800 | *CENPK2-1D MATa, ura3-52, leu2-3,112, trp1-289, his3Δ1, gal80::G418* |
| C104 | *C800 harboring the ZS104 plasmid* |
| C106 | *C800 harboring the ZS106 plasmid* |
| C137 | *C800 harboring the ZS137 plasmid* |
| C141 | *C800 harboring the ZS141 plasmid* |
| C142 | *C800 harboring the ZS142 plasmid* |
| C143 | *C800 harboring the ZS143 plasmid* |
| C144 | *C800 harboring the ZS144 plasmid* |
| C145 | *C800 harboring the ZS145 plasmid* |
| C159 | *C800 harboring the ZS159 plasmid* |
| C173 | *C800 harboring the ZS173 plasmid* |

**Table S2. Primers used in this study.**

| Primer | sequence |
| --- | --- |
| *Ag*F3H-up | ATGAACTCTTTGGCTTTGTTGTTCTGTAC |
| *Ag*F3H-down | GTCTCCACAAGCTTTCGGCAAGTAA |
| *Ag*F3′H-up | ATGAACTCTTTGGCTTTGTTGTTCTGT |
| *Ag*F3′H-down | GTCTCCACAAGCTTTCGGCAAGTAA |
| *Ag*F3′5′H-up | ATGGCTATTGATACATCATTGTTAGTTGAATTAGCT |
| *Ag*F3′5′H-down | CCAAGATTACATCAATCTGCTTATGCTGTTTAA |
| *Ag*CPR1-up | ACCTTCACAGAGCTCGATTGCAT |
| *Ag*CPR1-down | TCACCACACATCACGTAAATACCTCC |
| *Ag*CPR2-up | GTTAATCACATCTGATTCGGAAGCCAT |
| *Ag*CPR2-down | TCATACCTGCTCCTGGACAATGG |

**Table S3. Plasmids used in this study.**

| Plasmid ID | Relevant characteristics |
|---|---|
| ZS104 | pY26-GAL7p-*Ag*F3H-CYC1t |
| ZS106 | pY26-ADH1t-*Sm*CPR-TDH1p-INO1p-*Ag*F3′H-CYC1t |
| ZS137 | pY26-ADH1t-*Ag*CPR2-TDH1p-INO1p-*Ag*F3′H-CYC1t |
| ZS141 | pY26-ADH1t-*At*CPR-TDH1p-INO1p-*Ag*F3′H-CYC1t |
| ZS142 | pY26-ADH1t-*Eb*CPR-TDH1p-INO1p-*Ag*F3′H-CYC1t |
| ZS143 | pY26-ADH1t-*Ht*CPR-TDH1p-INO1p-*Ag*F3′H-CYC1t |
| ZS144 | pY26-ADH1t-*Gm*CPR-TDH1p-INO1p-*Ag*F3′H-CYC1t |
| ZS145 | pY26-ADH1t-*Sc*CPR-TDH1p-INO1p-*Ag*F3′H-CYC1t |
| ZS159 | pY26-ADH1t-*Ag*CPR1-TDH1p-INO1p-*Ag*F3′H-CYC1t |
| ZS173 | pY26-ADH1t-*Ag*CPR-TDH1p-INO1p-*Ag*F3′5′H-CYC1t |

**Table.S4. Fragment per kilo bases per million reads (FPKM) of different genes in every sample.**

| Gene name | LS1 | LS5 | Stem | Tendril | Root |
|---|---|---|---|---|---|
| *PAL1* | 67.9±2.30 | 17.8±0.96 | 60.1±0.71 | 94.2±4.16 | 189.6±37.51 |
| *PAL2* | 50.3±2.45 | 8.9±0.64 | 11.7±4.78 | 51.2±6.19 | 100.0±22.21 |
| *PAL3* | 253.3±9.06 | 51.3±1.22 | 27.6±11.05 | 145.5±14.52 | 187.1±3.66 |
| *C4H* | 5.4±0.54 | 6.6±0.33 | 0.16±0.16 | 2.8±2.74 | 4.5±2.58 |
| *4CL* | 107.2±3.68 | 36.2±1.56 | 17.2±2.66 | 69.1±5.08 | 55.4±0.87 |
| *CHS* | 1198.8±31.39 | 406.0±4.20 | 98.9±32.60 | 334.1±15.91 | 447.6±28.69 |
| *CHI* | 335.4±5.66 | 223.7±11.20 | 63.4±3.75 | 120.2±14.83 | 205.7±0.58 |
| *F3H* | 484.7±3.05 | 123.9±5.92 | 82.3±21.93 | 224.1±13.61 | 373.1±24.81 |
| *F3′H* | 183.4±6.39 | 59.6±3.33 | 40.1±6.14 | 93.9±3.77 | 146.4±14.48 |
| *F3′5'H* | 1036.7±26.78 | 416.6±17.33 | 343.7±81.48 | 639.0±16.46 | 155.17±38.92 |
| *CPR1* | 234.2±10.00 | 177.9±3.83 | 61.3±4.58 | 107.5±12.53 | 212.9±36.05 |
| *CPR2* | 14.8±0.69 | 13.6±1.14 | 14.7±0.41 | 14.7±0.45 | 26.1±2.92 |
| *DFR1* | 32.9±0.59 | 12.9±0.75 | 23.7±1.54 | 36.1±1.12 | 237.9±40.44 |
| *DFR2* | 0.42±0.16 | 0.355±0.13 | 11.3±0.66 | 0.7±0.10 | 42.4±2.83 |
| *FLS1* | 108.1±2.19 | 39.8±1.78 | 47.5±8.21 | 103.9±4.10 | 473.1±56.39 |
| *FLS2* | 244.4±9.08 | 96.1±4.44 | 0.6±0.12 | 5.0±0.47 | 0.33±0.02 |
